# Lung function trajectories in children with pulmonary TB and non-TB lower respiratory tract infections

**DOI:** 10.5588/ijtldopen.25.0080

**Published:** 2025-08-13

**Authors:** I.J. Courtney, M. Palmer, R. Swanepoel, C.J. Lombard, M. van Niekerk, R. Dunbar, E.D. McCollum, H.S. Schaaf, A. Gie, P. Goussard, A.C. Hesseling, V.W. Jongen, M.M. van der Zalm

**Affiliations:** ^1^ Desmond Tutu TB Centre, Department of Paediatrics and Child Health, Stellenbosch University, Cape Town, South Africa;; ^2^ Pulmonology Tygerberg Academic Hospital, Department of Pulmonology, Cape Town, South Africa;; ^3^ Division of Epidemiology and of Biostatistics, Department of Global Health, Stellenbosch University, Cape Town, South Africa;; ^4^ Biostatistics Research Unit, South African Medical Research Council, Cape Town, South Africa;; ^5^ Global Program in Pediatric Respiratory Sciences, Eudowood Division of Pediatric Respiratory Sciences, Department of Pediatrics, Johns Hopkins School of Medicine, Baltimore, United States;; ^6^ Department of Paediatrics and Child Health, Stellenbosch University, Cape Town, South Africa;; ^7^ Department of Infectious Diseases, Public Health Service Amsterdam, Amsterdam, The Netherlands;; ^8^ Stichting HIV monitoring, Amsterdam, The Netherlands;; ^9^ Amsterdam Institute for Immunology and Infectious Diseases (AI&I), Amsterdam, The Netherlands.

**Keywords:** tuberculosis, paediatric tuberculosis, spirometry, TB-associated respiratory morbidity, PTLD

## Abstract

**BACKGROUND:**

This longitudinal study compared lung function in children with pulmonary TB (PTB), children with non-TB lower respiratory tract infections (LRTIs) and healthy controls.

**METHODS:**

Children aged 4–13 years presenting with presumed PTB and their healthy siblings who could perform spirometry were included. Children were classified as having TB, non-TB LRTIs after careful evaluation and during follow-up. Spirometry measurements were completed at baseline and at subsequent study visits during 52 weeks of follow-up. Measurements included forced expiratory volume in 1 second (FEV_1_), forced vital capacity (FVC), and FEV_1_/FVC using 2022 race-neutral Global Lung Initiative reference ranges.

**RESULTS:**

Of 143 children, 46 had TB, 64 had non-TB LRTIs, and 33 were healthy controls. The median age was 6 years (IQR 5–9) and 10 (7%) were living with HIV. Restrictive spirometry patterns were common in both symptomatic groups at the end of follow-up, with a significantly lower FVC in children with TB compared to controls. In multivariable analysis adjusted for time and study group, FEV_1_ and FVC decreased for both the TB and non-TB LRTI groups, compared to healthy controls.

**CONCLUSION:**

Lung-function trajectories were similar between children with TB and non-TB LRTI, with low FVC one-year after diagnosis.

TB remains a significant global health challenge.^1^ It causes substantial morbidity and mortality, but there is also increasing evidence that, even after successful treatment, a substantial proportion of adults suffer long-term TB-associated respiratory morbidity, known as post-TB lung disease (PTLD).^2,3^ In 2023, a systematic review and meta-analysis with data from 14,621 people showed that 59% of adults who had spirometry after TB treatment completion had abnormal lung function, with 22% having obstructive, 23% having restrictive, and 15% mixed pattern lung impairment.^4^ Of the 155 million TB survivors alive in 2020, an estimated 15–19 million were children <15 years of age who may be at risk for long-term TB-associated respiratory morbidity.^5^ The pathophysiology of childhood pulmonary TB (PTB) differs from adult-type PTB, with less destructive cavitary lesions observed in young children.^6^

However, childhood TB represents a wide spectrum of disease regarding disease phenotypes and disease severity, often influenced by the age at presentation.^7^ Importantly, as children’s lungs are still developing, TB can impact the lung’s ability to reach their full functional potential in adulthood.^8–10^ Recent data indicate that PTB in childhood is associated with persistent respiratory symptoms, reduced quality of life (QoL), radiological abnormalities and lung function impairment following completion of TB treatment.^11–14^ Additionally, studies have shown that respiratory incidents, such as lower respiratory tract infections (LRTIs), can cause ‘de-tracking’ of lung function, where lung function declines after the event and fails to fully recover to pre-morbid levels.^15–17^ Limited data are available on the (differential) impact of PTB and other LRTIs on the long-term lung health of children. A recent study from South Africa indicated that PTB may cause greater impairment than non-TB LRTIs in children, although follow-up data beyond 3 months was unavailable.^18^

To address this knowledge gap, our study aimed to assess the long-term impact of PTB on spirometry measured lung function over a 12-month period, compared to children with non-TB LRTIs. We evaluated spirometry measurements across three groups: children with PTB (microbiologically confirmed and clinically diagnosed), symptomatic children in whom TB was excluded (non-TB LRTI), and healthy sibling controls.

## METHODS

### Study design and population

Participants were part of an ongoing, observational TB diagnostic and lung health cohort study (Umoya) which started recruitment November 2017, and study methods have been described elsewhere.^19^ In brief, children aged 0–13 years, with and without HIV, were eligible if they presented with presumptive PTB. A non-study clinician made the decision to start TB treatment (children with TB) or not (non-TB LRTI, also referred to as LRTI controls), and TB treatment was given within routine services according to National TB guidelines. In addition, we included healthy controls who were siblings of children enrolled in the study without chronic symptoms or underlying comorbidities associated with possible impaired lung function, but with similar environmental exposures. This was a convenience sample and they were not formally matched on age and sex. Healthy sibling controls were enrolled when identified and followed up with the same visit schedule.

### Study procedures

All children were carefully examined at enrolment and a medical history was taken, including details of previous TB episodes. Information on risk factors for poor lung health, such as HIV infection or exposure, family history of asthma and environmental factors including tobacco smoke exposure during and after pregnancy, were recorded. Routine TB investigations included the collection of two or more respiratory samples for microbiology, chest x-ray (CXR) and tuberculin skin test (TST). Concentrated smear microscopy (Auramine O), culture in BACTEC Mycobacteria Growth Indicator Tube 960 (MGIT 960) liquid medium (Becton Dickinson, MD), and Xpert MTB/RIF or Ultra (Ultra from 28 March 2018) were performed on all respiratory samples. Anterior-posterior (AP) or posterior-anterior (PA) digital CXRs were obtained depending on the participant’s age, as well as a lateral view. CXRs were read by two experts (HSS and PG) and classified as ‘normal’, ‘abnormal with features typical of TB’, or ‘abnormal with features not typical of TB’, and for radiological disease severity.^20^ Typical TB was classified as the presence of any of the WHO-defined CXR radiological features. If none of these features were present, the CXR was classified as not typical TB. Severe radiological disease was classified as the presence of cavities, miliary infiltrates, airway compression/deviation, complicated pleural effusion and/or consolidation involving ≥1 lobe. If the two readers disagreed on the certainty of TB disease, consensus agreement was obtained during a meeting.

### Spirometry

Spirometry was attempted in all children ≥4 years of age and was conducted at baseline, weeks 2, 8, 24, and 52. For this analysis we included all children with at least one spirometry measurement during 52 weeks of follow-up. Children who turned 4 years of age after their baseline visit were included in the analyses from their first spirometry measurement. The Jaeger spirometry was conducted with the child in a sitting position according to the European Respiratory Society (ERS)/American Thoracic Society (ATS) guidelines by a trained lung function technologist.^21^ The flow volume test was conducted with the child breathing normally through a mouthpiece. The child was instructed to inspire maximally followed by exhaling rapidly and forcefully continuing to complete exhalation until a plateau (volume change of <25ml) for 1 second was noted. Post-bronchodilator response was measured by comparing the flow volume curves before and 15–20 minutes after using a spacer to inhale 400 micrograms of a short-acting bronchodilator (salbutamol). Lung function measurements obtained were converted to z-scores using race-neutral Global Lung Initiative (GLI) reference range equation adjusted for age, gender and height.^22,23^ We only included lung function measurements that met the ERS/ATS quality criteria for acceptability and repeatability; for the analysis we used the best of three pre- and post-bronchodilation measurements.^21^ An obstructive spirometry pattern was defined as forced expiratory volume in one second (FEV_1_)/forced vital capacity (FVC) ratio less than -1.64 z-score (lower limit of normal (LLN)) with FVC>LLN; restrictive pattern as FVC < LLN and normal FEV_1_/FVC, and abnormal spirometry if there was an obstructive and/or restrictive spirometry pattern. Bronchodilator response was defined as an FEV1 increase of 10% or more in predicted value.

### Sample size

We did a repeated measures ANOVA sample size calculation for comparing the mean FEV1 z-score profiles between children with TB and healthy controls, based on adult data available at the time. We assumed there would be an improvement of +.2 on the z-scale, with a standard deviation of 1.25 from diagnosis to beyond treatment completion, which is a conservative measure as this was based on improvement after treatment completion. For the healthy group we assumed an FEV1 z-score of 0 at both time points with the same standard deviation of the TB group. With a significance level .05 and power at 90%, we needed 19 children per group and this was inflated to 23 per group to allow for a 20% loss to follow-up.

### Statistical Analysis

Baseline was defined as the enrolment visit or the start of TB treatment for children with PTB Follow-up continued until study discontinuation, loss-to-follow-up, or week 52. We compared baseline characteristics between children with PTB, LRTI controls, and healthy controls using Kruskal-Wallis non-parametric test for continuous data and Pearson’s Chi-squared test or Fisher’s exact test for categorical variables. Standardized z-scores for pre- and post-bronchodilator FEV_1_, FVC, and FEV_1_/FVC were reported as medians and interquartile ranges (IQRs) and compared between study groups using Kruskal-Wallis non-parametric test and Wilcoxon rank-sum test. The absolute number and proportion of children with TB and controls with an abnormal FEV_1_, FVC, FEV_1_/FVC, obstructive, restrictive and abnormal spirometry impairment were described, as well as post bronchodilator response. We used logistic regression to compare obstructive, restrictive, and abnormal lung function between groups and report odds ratios (OR) with 95% confidence intervals (CI).

We assessed the effect of time and study group on the standardized FEV_1_, FVC and FEV_1_/FVC z-scores using quantile regression with a robust cluster variance estimator to account for repeated measurements within a participant. To compare the median profile of the outcomes between the study groups, we tested an interaction effect between time and study group. We estimated the predicted median change over time between study groups using the ‘margins’ command in STATA. Analyses were conducted in STATA (v17.0, StataCorp, College Station, TX, USA). A significance level of 5% was used for testing the study hypothesis.

### Ethics statement

Stellenbosch University Health Research Ethics Committee approved the study (HREC N17/08/083). Informed consent was obtained from parents or legal guardians and in addition, assent from children ≥7 years prior to enrolment.

## RESULTS

Between 22 November 2017 to 1 December 2024, 622 children (0–13 years) were enrolled. Of these, 372 (59.8%) children aged ≥ 4 years had at least one spirometry measurement, of whom 172 children were excluded because measurements did not meet quality criteria for acceptability and repeatability. Thus, 143 children were analysed; 46 children with PTB, 64 LRTI controls (non-TB LRTI), and 33 healthy controls (Supplementary Data Figure S1). Of children treated for TB, 18/46 (39.1%) were microbiologically confirmed. The median age at baseline was 6 years (interquartile range [IQR] 5–9) and did not differ between study groups (p=0.829, Table 1). Of the 143 participants, 76 (53%) were male, 24 (20%) were born premature (gestational age <37 weeks), 17 (14%) were HIV exposed uninfected, and 10 (7%) were living with HIV. Previous TB treatment was reported for 22 (15%) children and 27 (19%) children were previously admitted to hospital due to non-TB LRTI. Exposure to smoking during pregnancy and within the household postnatally was prevalent across all groups. The most frequently reported symptoms were cough, fever, lack of appetite, and failure to thrive (Table 2).

**Table 1.** Baseline characteristics of all included children.

	Total (n=143) *n(%)*^A^	Children with TB (n=46) *n(%)*^A^	Non-TB LRTI (n=64) *n(%)*^A^	Healthy controls (n=33) *n(%)*^A^	*p-value*
Males	76 (53%)	25 (54%)	35 (55%)	16 (48%)	0.829
Age (yrs), median (IQR)	6 ([5-9])	6 [5-9]	6 [5-8]	7 [5-9]	0.756
Height for age (z-score), median [IQR]	-1.01 [-1.61, -0.40]	-1.00 [-1.75, -0.44]	-1.00 [-1.61, -0.50]	-1.01 [-1.33, -0.24]	0.409
BMI for age (z-score), median [IQR]	-0.46 [-1.08, 0.22]	-0.45 [-1.23, 0.34]	-0.48 [-1.04,0.20]	-0.30 [-0.87,0.22]	0.894
Born premature^B^	24 (20%)	8 (21%)	10 (18%)	6 (21%)	0.945
HIV exposed, uninfected^C^	17 (14%)	6 (15%)	6 (11%)	5 (19%)	0.633
Living with HIV	10 (7%)	4 (9%)	5 (8%)	1 (3%)	0.586
**Medical history n (%)**					
Hospital admissions for LRTI	27 (19%)	11 (24%)	14 (22%)	2 (6%)	0.108
Previous TB treatment	22 (15%)	6 (13%)	12 (19%)	4 (12%)	0.600
Family history of asthma, allergies, eczema	71 (50%	20 (43%)	36 (58%)	15 (45%)	0.264
Maternal history of asthma, allergies, eczema^D^	37 (52%)	10 (50%)	20 (56%)	7 (47%)	0.825
Paternal history of asthma, allergies, eczema^D^	9 (13%)	2 (10%)	5 (14%)	2 (13%)	0.913
**Potential insult to lungs**					
Maternal smoking during pregnancy	64 (46%)	18 (40%)	35 (56%)	11 (37%)	0.135
Smoking exposure after birth	101 (72%)	30 (67%)	47 (73%)	24 (75%)	0.661
Indoor cooking smoke (fire/paraffin) exposure	6 (4%)	2 (4%)	3 (5%)	1 (3%)	0.936
**Social economic circumstances**					
Unemployed in household	55 (39%)	12 (26%)	28 (44%)	15 (45%)	0.110
Grants received	124 (87%)	38 (83%)	56 (89%)	30 (91%)	0.485
Type of housing (brick)	103 (72%)	34 (74%)	50 (78%)	19 (58%)	0.096
Type of housing (informal)	40 (28%)	12 (26%)	14 (22%)	14 (42%)	0.096

Data were missing for: Born premature (n=20), HIV exposure (n=10), hospital admissions for LRTI (n=1), family history of asthma, allergies, eczema (n=2), maternal smoking during pregnancy (n=5), smoking exposure after birth (n=2), indoor cooking smoke (fire/paraffin) exposure (n=1), grants received (n=1).

A Unless otherwise specified;

B Defined as a gestational age of <37 weeks;

C Among those without HIV (n=143);

D Among those with a family history of asthma, allergies or eczema.

**Table 2.** Baseline symptoms of children classified as children with PTB and non-TB LRTI.

	Children with TB (n=46)	Children with non-TB LRTI (n=64)	
	*n (%)*	*n (%)*	p-value
**Cough**	40 (87)	61 (95)	0.161
**Pattern of cough** ^A^			0.376
- Acute	15 (38)	19 (32)	
- Chronic (>2wks)	24 (60)	36 (59)	
- Recurrent	1 (3)	6 (10)	
Wheeze	13 (28)	16 (25)	0.827
Fever	21 (46)	30 (47)	1.000
Lack of appetite	26 (57)	24 (38)	0.055
Failure to thrive	21 (49)	25 (42)	0.548
Lethargic	6 (13)	3 (5)	0.161
Night sweats	18 (39)	21 (33)	0.547
**CXR finding**			
Abnormal	19 (59)	16 (29)	0.005
Typical TB^B^	12 (67)	2 (13)	0.004
Severe TB^C^	9 (82)	1 (50)	0.423

Data missing for: failure to thrive (n=7), abnormal CXR (n=23), typical TB (n=2), severe TB (n=1).

A Among those who reported a cough;

B Among those with an abnormal CXR reading;

C Among those with typical TB on CXR

CXR imaging showed typical CXR features in 14 children, 12 in children treated for TB and 2 in LRTI controls. Of the children treated for TB, 9/46 (20%) had severe PTB. The CXRs of the two LRTI controls with features typical for TB were re-evaluated and considered to have non-TB LRTI on clinical case review.

### Lung function

Cross-sectional analysis of spirometry baseline measurements showed evidence towards lower post-bronchodilation FEV_1_ in children with TB (median=-0.43, IQR=-1.60;0.40) and in LRTI controls (median=-0.58, IQR=-2.19;-0.23) compared to healthy controls (median=1.11, IQR=-1.83;1.79, p=0.049). Similarly, FVC was lower in children with TB (median=-0.91, IQR=-2.04;-0.09) and LRTI control children (median=-0.81, IQR=-1.40;0.11) compared to healthy controls (median=0.37, IQR=-0.43;0.87, p=0.024) (Table 3). At the 52-week follow-up, children with TB had significantly lower FVC compared to controls, both non-TB LRTI and healthy controls. Table 4 shows the spirometry patterns in children with TB, LRTI controls and in healthy controls during follow-up. None of the children had a mixed spirometry impairment. Restrictive spirometry patterns were most common in children with TB and in LRTI controls at the end of follow-up. Bronchodilator response was seen in 20 children, without differences between groups. In multivariable analysis including time of assessment and study group, we found that FEV_1_ and FVC slightly decreased over time for children with TB and in LRTI controls compared to healthy controls, albeit not significant (Figure; Supplementary Data Table S1).

**Table 3.** Spirometry Z-score data of healthy controls, Non-TB LRTI and Children with TB pre- and post-bronchodilation, Cape Town, South Africa, April 2018–March 2025.

	Children with TB	Non-TB LRTI	Healthy controls		Children with TB vs. Non-TB LRTI	Children with TB vs. healthy controls
	*Median (IQR)*	*Median (IQR))*	*Median (IQR)*	*Overall p-value*	*p-value*	*p-value*
**Baseline**						
*Pre- bronchodilation*	(n=29)	(n=46)	(n=24)			
FEV_1_	-0.22 (-1.44-0.43)	-1.04 (-2.23-0.07)^A^	-0.69 (-2.29-1.22)	0.480	0.275	0.780
FVC	-0.81 (-1.93-0.09)	-1.04 (-1.76-0.31)	0.27 (-1.28-0.72)	0.064	0.598	**0.020**
FEV_1_/FVC	1.38 (1.30-1.86)	1.55 (0.97-1.97)^A^	1.39 (-1.02-2.18)	0.950	0.769	0.823
*Post bronchodilation*	(n=21)	(n=34)	(n=15)			
FEV_1_	-0.43 (-1.60-0.40)	-0.58 (-2.19-0.23)	1.11 (-1.83-1.79)	**0.049**	0.869	**0.039**
FVC	-0.91 (-2.04-0.09)	-0.81 (-1.40-0.11)	0.37 (-0.43-0.87)	**0.024**	0.203	**0.009**
FEV_1_/FVC	1.59 (1.31-2.09)	1.50 (0.97-1.83)	2.17 (1.36-2.26)	0.100	0.323	0.136
**Week 24**						
*Pre- bronchodilation*	(n=30)	(n=39)	(n=19)			
FEV_1_	-0.49 (-1.57-0.27)	-1.00 (-2.31-0.00)	-1.83 (-2.61-0.30)	0.361	0.590	0.246
FVC	-0.77 (-1.41-0.06)	-0.68 (-1.54-0.44)	-0.35 (-2.42-0.60)	0.735	0.479	0.798
FEV_1_/FVC	1.44 (1.22-1.83)	1.42 (-3.51-1.80)	1.59 (-2.74-2.13)	0.514	0.387	0.918
*Post bronchodilation*	(n=21)	(n=26)	(n=13)			
FEV_1_	-0.10 (-1.44-0.27)	-0.94 (-1.69-0.22)	-0.43 (-1.83-1.09)	0.610	0.507	0.535
FVC	-0.60 (-1.13-0.19)	-0.64 (-1.68-0.44)	-0.27 (-1.41-1.07)	0.907	0.957	0.583
FEV_1_/FVC	1.40 (1.19-1.61)	1.51 (1.10-1.86)	1.65 (0.92-2.19)	0.599	0.563	0.425
**Week 52**						
*Pre- bronchodilation*	(n=25)	(n=36)	(n=16)			
FEV_1_	-1.03 (-1.62-0.21)	-0.89 (-1.62-0.40)	0.74 (-1.21-1.71)	0.160	0.583	**0.027**
FVC	-1.06 (-2.18-0.81)	-0.94 (-1.81-0.21)	0.36 (-0.86-0.91)	**0.009**	0.215	**0.003**
FEV_1_/FVC	1.66 (1.35-2.00)	1.59 (1.17-1.91)	1.56 (0.81-2.26)	0.610	0.284	0.728
*Post bronchodilation*	(n=22)	(n=26)	(n=12)			
FEV_1_	-1.16 (-2.00-0.22)	-0.97 (-1.86-0.05)	1.18 (-1.48-1.74)	0.068	0.321	0.040
FVC	-1.39 (-2.18-0.81)	-1.08 (-1.91-0.27)	0.43 (-0.11-1.22)	**<0.001**	0.196	**<0.001**
FEV_1_/FVC	1.66 (1.34-1.97)	1.69 (1.22-1.96)	1.16 (-1.43-2.22)	0.745	0.975	0.407

A 1 missing. FVC = forced vital capacity; FEV_1_ = forced expiratory volume in 1 second; MEF_25_ = maximum expiratory flow at 25% FVC; SDstandard deviation.

**Table 4.** Spirometry patterns of healthy controls, symptomatic controls (non-TB LRTI) and children with PTB pre- and post-bronchodilation, Cape Town, South Africa, April 2018–March 2025.

	Total abnormal		Obstructive		Restrictive	
	*n (%)*	*OR (95%CI)*	*n (%)*	*OR (95%CI)*	*n (%)*	*OR (95%CI)*
**Baseline**						
Healthy controls	10 (42%)	REF	6 (25%)	REF	4 (17%)	REF
Non-TB LRTI	22 (48%)	1.28 (0.47-3.48)	9 (20%)	0.73 (0.23-2.37)	13 (28%)	1.97 (0.56-6.88)
Children with TB	11 (38)	0.86 (0.28-2.58)	1 (3%)	0.11 (0.01-0.97)	10 (34%)	2.63 (0.70-9.84)
**Week 24**						
Healthy controls	10 (53%)	REF	5 (26%)	REF	5 (26%)	REF
Non-TB LRTI	19 (49%)	0.86 (0.29-2.56)	10 (26%)	0.97 (0.28-3.37)	9 (23%)	0.84 (0.24-2.97)
Children with TB	10 (33%)	0.45 (0.14-1.46)	3 (10%)	0.31 (0.06-1.50)	7 (23%)	0.85 (0.23-3.21)
**Week 52**						
Healthy controls	4 (25%)	REF	3 (19%)	REF	1 (6%)	REF
Non-TB LRTI	14 (39%)	1.91 (0.51-7.11)	4 (11%)	0.54 (0.11-2.67)	10 (28%)	5.77 (0.67-49.61)
Children with TB	11 (44%)	2.36 (0.59-9.37)	1 (4%)	0.18 (0.02-1.92)	10 (40%)	10.00 (1.13-88.917)

**Figure.**
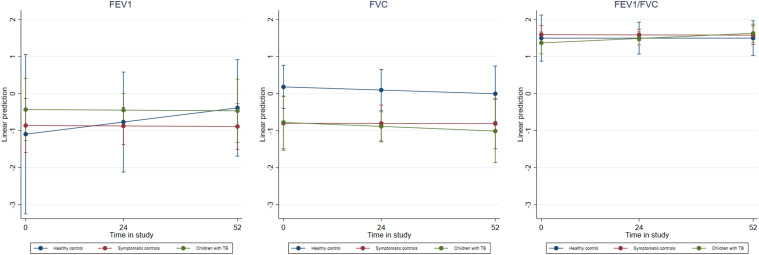
Estimated means over time of FEV1, FVC and FEV1/FVC in children with PTB, non-TB LRTI and healthy controls during follow-up. FEV_1_, FVC, FEV_1_/FVC over time based on the unadjusted quantile regression models. The marginal predicted mean of FEV_1_, FVC, and FEV_1_/FVC did not change significantly over time for any of the study populations. FVC = forced vital capacity; FEV_1_ = forced expiratory volume in 1 second; LRTI = lower respiratory tract infection.

## DISCUSSION

We found similar lung function trajectories between children with PTB and children with non-TB LRTI compared to healthy controls, with mainly a low FVC that worsened one-year after diagnosis. Although there were no significant differences in FEV_1_ between the groups, there was a trend towards a decline in FEV_1_ in children with TB and non-TB LRTIs compared to healthy controls during 52 weeks of follow-up. Our findings underscore the impact of respiratory illnesses, including PTB, on lung function. The majority of data on the impact of LRTIs on lung function in children are derived from high-income countries.^15,17,24,25^ These studies consistently demonstrate that children who experienced an LRTI tend to have lower lung function compared to those who did not. Similar findings have been reported in some African studies, where the burden of both TB and LRTIs is high.^26,27^ In our study, the median FEV_1_ and FVC was below a z-score of 0 in all children with TB and non-TB LRTI. While a z-score between +1.64 and -1.64 is considered normal, research has shown that even a subclinically reduced FEV_1_ and FVC is associated with a higher risk of respiratory and cardiovascular events later in life.^28^ The observed reduction in lung function among children with TB and those with non-TB LRTIs may therefore have substantial long-term consequences.

Low FVC, pointing towards a possible restrictive lung function pattern was common in our study, which is consistent with findings from other paediatric studies examining lung function following TB and studies among adults.^4,11,12,14,18^ In contrast, recent data from a similar cohort in Cape Town demonstrated improved FVC trajectories over the same follow-up period, with decreasing trajectories for FEV_1_/FVC in children with confirmed TB, compared to children with clinically diagnosed TB and children with non-TB LRTI. This finding suggests the development of possible obstructive disease in children with confirmed TB.^18^ In our study we did not observe changes in FEV_1_/FVC ratio among children with TB, and all three groups generally maintained above-normal z-scores. The differences between these studies can possibly be explained by the differences in spectrum and severity of disease of the children included. Additionally, our data show an overall worsening of FVC over time both in children with TB and non-TB LRTIs compared to healthy controls, which could be explained by delayed recovery of lung function due to ongoing inflammation^29^ or underlying comorbidities impacting on lung health.^8,30^ Although low FVCs are consistently reported in children that completed TB treatment,^11,12^ including studies that measured volumes,^14^ technical issues associated with low FVCs need to be excluded. Further research is essential to establish long-term lung function trajectories in these children beyond childhood and to explore how specific features of paediatric TB, such as hilar lymph node disease, cavities, pleural effusions, and parenchymal involvement, influence lung health outcomes.

TB is a disease of poverty and all children enrolled in this study generally come from low socio-economic backgrounds with associated high prevalence of risk factors (e.g., smoking, HIV, previous TB) associated with impaired lung health.^8,31,32^ Although there have been some concerns about the use of race-neutral reference ranges in sub-Saharan African populations,^33,34^ our analysis was primarily focused on comparing differences between symptomatic children and local healthy controls. Our findings indicate that the race-neutral reference ranges provide an accurate representation of lung function in healthy children from this setting. The majority of children in this study were stunted, which is likely a reflection of chronic poor nutrition.^35^ Previous research has shown that stunting has an association with poorer lung function and lung growth.^31^ The impact of stunting on overall lung health warrant further investigations. Other risk factors for poor lung function include smoking, both active and passive, previous respiratory illness, HIV infection and family history of asthma.^36,37^ Overall, these risk factors were comparable between our study groups, including healthy controls, which may have contributed to the overall poorer lung function outcomes. Maternal smoking during pregnancy and household smoking exposure after birth in this cohort was high and strategies to address these risk factors should be a public health priority. A high proportion of children in our cohort had been previously treated for TB, which is a reflection of the high burden of TB seen in this young population. An incremental effect of TB episodes on lung function has been described and further studies are needed to investigate this phenomenon in children and adolescents.^38^

The strengths of this study include the careful assessment and long-term follow-up of children with TB, non-TB LRTI and healthy controls. The healthy controls were siblings of children with presumptive TB and thus have a similar socio-economic status and exposure to environmental and social factors influencing lung health, making them a robust comparison group. Limitations include the small sample size which precluded sub-group analyses and the ability to detect small differences. The spectrum of TB-associated respiratory morbidity is likely to vary according to spectrum and severity of disease. As previously shown, the majority of children with TB also have other respiratory pathogens (such as viruses), which likely will have an impact on lung function in both children with TB and non-TB LRTIs.^39^ Furthermore, the highest burden of paediatric TB occurs in children under the age of 5 years who are generally unable to perform spirometry. Pre-school lung function measurements including oscillometry and other tidal breathing techniques should be considered to track lung function longitudinally across this important young age group.

## CONCLUSION

This study highlights the significant impact of TB and non-TB LRTIs on long-term lung health in young children. Overall, these findings underscore the need to prioritize early prevention and treatment. Reducing harmful exposures like smoking is essential to improve outcomes.

## Supplementary Material


