# Supplementary material for: Lung function trajectories in children with pulmonary TB and non-TB lower respiratory tract infections
Source: IJTLD Open. 2025 Aug 13;2(8):471–7. doi: 10.5588/ijtldopen.25.0080 (PMC12352954; doi:10.5588/ijtldopen.25.0080)
Supplement: Supplementary file 1 [file ijtldopen25-0080_supplementarydata1.pdf]

Supplemental Table 1: Association between time and spirometry for children with TB, Non-TB LRTI, and healthy controls.[1]

	β^2	95% CI	p-value	Adjusted β^3	95% CI	p-value
**FEV1**						
Time in weeks	0.014	-0.03- 0.06	0.566	0.01	(-0.03-0.05)	0.623
Study arm						
Healthy controls	REF			REF		
**Non-TB LRTI**	0.23	-2.05-2.52	0.840	0.01	(-1.70-1.72)	0.988
Children with TB	0.66	-1.66-2.99	0.573	0.61	(-1.36 -2.58)	0.543
Study arm vs. time						
Healthy controls	REF			REF		
**Non-TB LRTI**	-0.01	-0.06-0.04	0.578	-0.01	(-0.05-0.03)	0.604
Children with TB	-0.01	-0.07-0.04	0.606	-0.01	(-0.06-0.03)	0.583
**FVC**						
Time in weeks	-0.004	-0.02-0.01	0.616	-0.007	(-0.03-0.02)	0.517
Study arm						
Healthy controls	REF			REF		
**Non-TB LRTI**	-0.968	-1.92- -0.048	0.039	-0.69	(-1.87-0.50)	0.255
Children with TB	-0.96	-1.88- -0.40	0.0.42	-0.71	(-2.09-0.67)	0.312
Study arm vs. time						
Healthy controls	REF			REF		
**Non-TB LRTI**	0.003	-0.02-0.03	0.779	0.008	(-0.02-0.04)	0.577
Children with TB	-0.001	-0.03-0.03	0.951	-0.0009	(-0.04-0.03)	0.963
**FEV1/FVC**						
Time in weeks	0.000	-0.01- 0.01	1.000	0.001	(-0.02-0.02)	0.914
Study arm						
Healthy controls	REF			REF		
**Non-TB LRTI**	0.09	-0.58-0.76	0.786	0.13	(-0.57-0.83)	0.722
Children with TB	-0.13	-0.82-0.56	0.714	-0.06	(-0.79-0.67)	0.870
Study arm vs. time						
Healthy controls	REF			REF		
**Non-TB LRTI**	-0.0002	-0.02-0.02	0.975	-0.004	(-0.03-0.02)	0.685
Children with TB	0.005	-0.01-0.02	0.535	0.001	(-0.02-0.02)	0.929

Abbreviations: FVC, forced vital capacity; FEV_1, forced expiratory volume in 1 second; 95% CI, 95% confidence interval; TB, tuberculosis

1. Results based on multivariable quantile regression
2. Multivariable model including time, study arm and a one-way interaction between time and study arm
3. Additionally adjusted for HIV status, maternal smoking during pregnancy, smoking exposure after birth, and BMI for age (z-score)

Supplemental Figure 1- Flowchart of children included in this study.

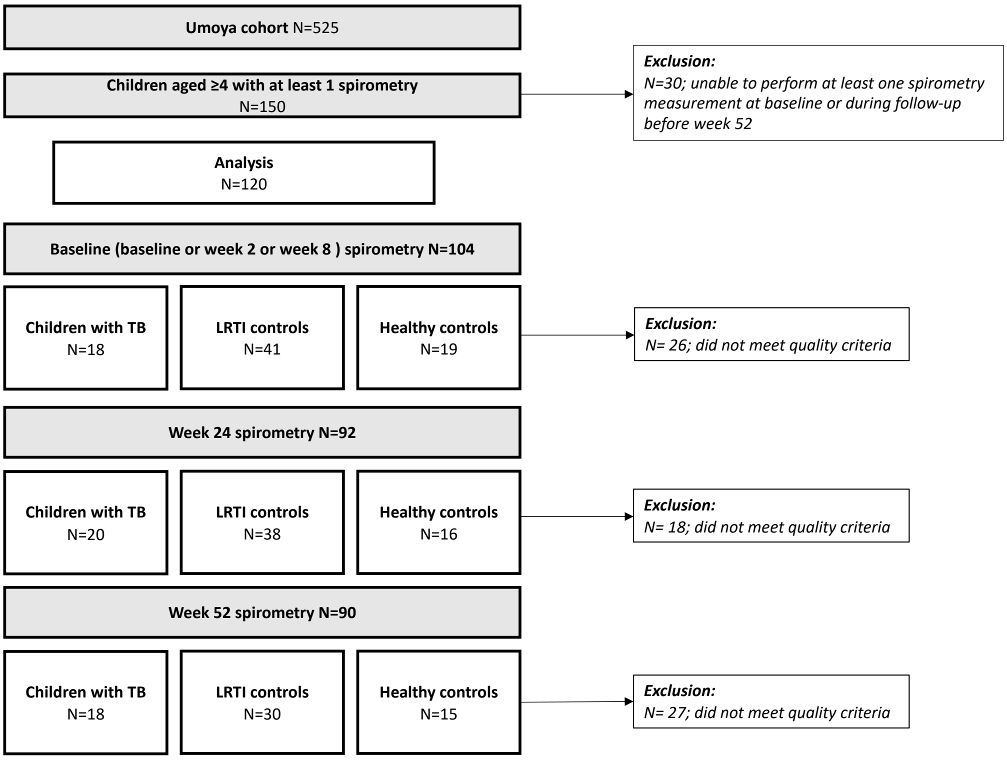

Legend: A total 200 children had at least 1 spirometry measurements at baseline or during follow-up before week 52. Lung function that did not meet quality criteria for repeatability and acceptability according to ERS/ATS guidelines were removed from the data-analysis, but if they had an acceptable quality lung function measurement at the next visit this data was analyzed.
